# Mr. Dennett, on Vaccination

**Published:** 1803-04-01

**Authors:** C. Dennett

**Affiliations:** Soho Square


### To JOHN RING, Esq.

SIR,

WHEN you did me the honour of applying for my signature of approbation on Vaccine Inoculation, I informed you, that, at that time, I had not had sufficient experience in it to make up my mind upon the subject; but having now practised it to very considerable extent, and with the most complete success, I feel it a duty due to the public and you, Sir, (for the unwearied attention paid in investigating this subject) to acknowledge now, my fullest confidence in it, and shall beg leave to trouble you with some observations, and at the same time recite a case or two.

At the early commencement of Vaccine Inoculation, I confess, I did not think it would ever supercede the Variolous; in this, I perhaps might be biassed by the great success which had attended a very extensive inoculation for the Small-pox, having inoculated, and seen treated by my father, between six and seven thousand patients, therefore, it cannot appear a matter of wonder if at first I was sceptical in this instance. And those parents who had witnessed the mildness of the disease under my particular treatment, would not permit me to use the Vaccine. But I feel a satisfaction in declaring, that there is not a comparison to be made, even under the mild form I have happily experienced. As a proof of which I vaccinated my last child, and strenuously endeavour to persuade every parent to have it used, but cannot always prevail. The greater part I have inoculated again with Small-pox matter, but in no instance have produced more than a slight local inflammation, for a few days, except in the following.

On September 26, 1801, I inoculated the daughter of Mrs. Snow, of Charlton Street, about two years and a half old, and did it in both arms, the one by puncture, the other by incision, with variolous matter taken from the arm of my own child on the eighth day, (at which period I conceive the virus to be most active.) On the 30th both arms were inflamed, but the one by incision most so. The 2d of October, Dr. Boys went with me to see the child; he agreed with me, that the inflammation (on the incised arm) was too violent for the time, and would subside without any fever. On the 4th, I found the child very feverish, the pulse being 130, and was informed she had been unwell since noon the day before. Dr. Boys saw the child again this day, as did Dr. M'Cammon, (now officiating at the Dispensary, Charles Street, Westminster,) and another medical gentleman. I believe they were all of opinion that an eruption would take place; but, on the 5th, the child was better, with general exanthemata over it; upon the thighs it had the appearance of urticaria. I did not give any medicine, being determined not to interfere, but understood the nurse had supplied the child with (their favorite remedy) saffron tea; being convinced, as she said, that it would have the Small-pox. On the 6th, the fever was quite gone; the inflammation upon the incised arm declining. On the 7th, there was great plenty of matter in both arms. I took some on quills. Dr. Boys wished for a quill, but as he did not send for it, I conclude he had not a patient at that time, on whom he would like to make trial of it; but Dr. M'Cammon used some, and produced a mild Small-pox with it. He informed me, that two or three were inoculated from his patient, who had the disease in a favorable manner. Though the inflammation in this case was of longer duration than usual, and produced the most unfavourable apprehensions, yet it is a satisfactory one, as it tends to prove, in the most incontrovertible manner, the impossibility of producing Small-pox eruption after Vaccine Inoculation. I must observe, that the child was vaccinated on November 22, 1800, and that the areola was later in forming than in general, not beginning before the eleventh day. Two children of a Mr. Hoskins were inoculated at the same time from my child, and in both, the inflammation continued until the seventh day, when it went off without any symptom of fever. They were also vaccinated twelve months before. I am happy in having an opportunity of stating a case, where I infected a child with the vaccine matter, when I could not with variolous. Every practitioner must have met with cases, when, under some particular constitution, the habit is not susceptible of the disease, either by infection or inoculation; culation; I have of late met with two instances in the same family.

In August, 1800, Mr. ———, had two children at the same time confined with the confluent Small-pox, one of whom died. An infant of three weeks old, was exposed to the infection the whole time, being always in the same room, and sometimes bed. I could not persuade the parents to have the child inoculated at that time, and to the Vaccine they positively objected. It did not receive the infection, and on the 7th of December following, was inoculated with fresh variolous matter without effect, and this was repeated three times with no better success. The father then declared himself satisfied, that the child must have received the infection naturally; I endeavoured to convince him to the contrary, and wished to insert matter again in May last, but he refused. On the 20th of last August, the mother was confined again. The former child being at a friend's, but, on the 27th was sent home ill, with symptoms of the Small-pox, which proved to be the fact, and were confluent in the face, but he recovered. The parents were now determined to have the infant inoculated with the Small-pox, but wished to have it done from their own child; therefore, as soon as matter could be procured from the face, it was applied by two punctures in the same arm, but it did not succeed. In three days after, I made use of the whole contents of one pustule, taken from the breast; having inserted it by puncture, as also by incision, this failed likewise. I then took matter from a fine pustule on the leg, just upon the turn; with this I was not more fortunate. The matter in this case was only carried from the bed to the window before used. This child also escaped the disease naturally, though the brother always slept in the bed with it. On the 12th of November, I inserted variolous matter again, without producing the desired effect. Being so often baffled, and having witnessed the sufferings of the former children, I thought myself justified in making use of vaccine matter, (though contrary to the parent's order) to try if the constitution would receive that. I have the gratification to add that it completely succeeded the first time. The parents did not suspect what I had done, but the nurse calling one day, told them it looked like the Cow-pox. The matter employed was a month old, kept on a quill, and used as I stated in No. 31, of the Medical and Physical Journal for September, 1801; you perhaps, Sir, did not see the description I there gave of my method, but in the number for the present present month, a statement very similar is given by you. Since that information was published, I have sent vaccine matter to Ireland, and several friends in the country, and have received the most flattering testimony of its utility. This I know, from two years experience, that, every gentleman has it in his power of always being provided with a certainty of keeping it active, by the means there described. In some instances I have sealed the end of the quills beside enclosing them securely in paper. Fourteen months back I inoculated in Baker Street, a patient of Dr. Boys, with variolous matter kept a considerable time, to shew him the manner I employed it; he expressed himself perfectly satisfied.

I have extended this letter to a much greater length than was at first intended, but trust it will be conceived to have arisen from the laudable motive of giving information upon a subject wherein the lives of so many thousands are concerned. And am, &c.

*Soho Square, Jan.* 22, 1803. C. DENNETT.

